# Structure-Function Correlations of Commercial Fucoidan Extracts: Antioxidant, Antiviral, Antifungal, Antibacterial and Prebiotic Activities

**DOI:** 10.3390/molecules31101618

**Published:** 2026-05-11

**Authors:** Matthew Chadwick, Maria Sole Regina Lancerin, Patricia Hazelton, Kyriakos Vidalis, Emmanuel Petit, Paolina Lukova, Cédric Delattre, Xianfeng Chen, Thamarai Schneiders, Vasso Makrantoni, Richard Sloan, Simone Dimartino

**Affiliations:** 1 Institute for Bioengineering, The School of Engineering, The University of Edinburgh, Edinburgh EH9 3JL, UK; 2 Centre for Inflammation Research, Institute of Regeneration and Repair, The University of Edinburgh, Edinburgh EH16 4UU, UK; 3 UMRT INRAe 1158 BioEcoAgro-BIOPI, IUT-GB, Université de Picardie Jules Verne, 80025 Amiens, France; 4 Department of Pharmacognosy and Pharmaceutical Chemistry, Faculty of Pharmacy, Medical University of Plovdiv, Vasil Aprilov Str. 15A, 4002 Plovdiv, Bulgaria; 5 Institut Pascal, CNRS, Clermont Auvergne INP, Université Clermont Auvergne, 63000 Clermont-Ferrand, France; 6 Institut Universitaire de France (IUF), 1 Rue Descartes, 75005 Paris, France; 7 Zhejiang University-University of Edinburgh Institute, Zhejiang University School of Medicine, Zhejiang University, Haining 314400, China; 8 Planet Crafting Labs, Edinburgh EH10 4AX, UK

**Keywords:** fucoidan, bioactivity, antioxidant, antiviral, antifungal, antibacterial, prebiotic, structure, structure–activity relationship, sulfation degree

## Abstract

Fucoidan is a sulfated polysaccharide derived from brown seaweed, reported to possess diverse biological activities that make it a molecule of great interest for nutraceutical and biomedical applications. A significant challenge to its wider implementation is a lack of understanding of the relationship between fucoidan’s structural and chemical characteristics with its biological activity. So far, approaches to identifying these relationships have been limited to qualitative comparisons of chemical and biological datasets or through chemically modified fucoidans. This work aimed to apply a formal methodology to elucidate potential relationships worthy of further exploration. The biological activity of commercial fucoidan extracts was assessed after detailed chemical characterization. The extracts exhibited multiple bioactivities, notably antioxidant activity, antiviral activity against Nipah virus, antifungal activity against *Candida dubliensis* and prebiotic effects on *Lactobacillus casei*, with no antifungal activity against *Candida albicans*, *Candida auris* and *Cryptococcus neoformans*, nor antibacterial effects against *Klebsiella pneumoniae*. Correlation analysis of biological activity and extract chemical characterization data identified several potential key quality attributes. Other than high fucose content, high sulfate content is identified as potentially important for antioxidant, antiviral, antifungal, and prebiotic activities. This work addressed the literature’s debate regarding the optimal molecular weight for bioactivity, suggesting that it depends on the specific microbe to which a fucoidan extract is applied. This study demonstrated that a formalized comparative approach, linking chemical and structural data with biological activity, can effectively identify important characteristics of fucoidan for a specific bioactivity. Future work will focus on expanding this approach by assessing the bioactivity of a wider array of chemically characterized fucoidan extracts. Additionally, extracts possessing identified quality attributes should be produced and employed in mechanistic bioactive studies to further validate the correlations drawn in this work.

## 1. Introduction

Fucoidan is a sulfated anionic polysaccharide found in the cell wall of brown seaweeds [1], with fucoidan reported to possess several bioactive properties, including antioxidant [2,3,4], antiviral [5,6,7], antifungal [8,9], anticancer [2,10], anticoagulant [11], antibacterial [12,13] and prebiotic activities [14]. The polysaccharide in seaweed contains substantial amounts of L-fucose [1] and several other monosaccharides, including xylose, rhamnose, galactose and mannose, as well as uronic acids, including glucuronic and galacturonic acid [15]. When fucoidan is extracted from seaweed, this complexity increases with a vast number of possible fucoidan structures (e.g., linear, branched) and a wide range of molecular weights (10 to 10,000 kDa) [16,17,18,19,20], plus additional contaminants from other cell wall components such as polysaccharides, proteins, and polyphenols.

The fucoidan structure and extract composition depend on many variables broadly grouped into two categories: environmental and process factors [15]. Environmental factors, such as seaweed species, harvesting geolocation, and season, affect the composition of the starting seaweed material before processing. Process factors, including the pre-treatment methods employed, the extraction technique and conditions used, and the purification methodology utilized, directly impact the composition and quality of the resulting fucoidan.

A major challenge to the commercialization of fucoidan as a bioactive ingredient is the poorly established relationship between the extraction process used and the resulting fucoidan’s chemical composition. The attributes a fucoidan should possess for a given biological activity remain unclear, and this relationship must be well characterized to facilitate fucoidan’s application in the nutraceutical and pharmaceutical markets.

So far, structure-bioactivity relationships have been investigated using several different approaches, including qualitative visual correlations [21], reducing the problem to a single seaweed source or processing method [22] or applying destructive unnatural chemical modification of fucoidan extracts [4]. While some factors important for bioactivity have been identified (monosaccharide profile, molecular weight, and sulfate content) [21], these approaches do not follow a formalized approach and rely on comparisons between two or more datasets.

This work aimed to address this research gap and elucidate potential key quality attributes associated with a given bioactivity for further exploration. This was achieved by selecting a set of commercially available fucoidan extracts reported to possess several biological activities covering a breadth of chemical and biological properties arising due to a wide variety of extraction processes, seaweed species, and geolocations. The extracts underwent detailed chemical and structural characterization (CHNS elemental analysis, monosaccharide profile, phenolic content, and molecular weight analysis) to elucidate their chemical structure and characteristics before bioactivity assessments. The results of these tests were then correlated using a formal Pearson’s correlation analysis, expanding the scope of previous analysis [23] to include antioxidant, antiviral, antifungal and prebiotic biological activities. This elucidated potential characteristics that a fucoidan should possess for improved bioactivity. Due to the small sample size, these findings require verification through further study and should only be interpreted as exploratory. Yet, this work provides a new workflow for identifying potential key quality attributes required for specific fucoidan biological activities.

## 2. Results

Fucoidan chemistry, structure and composition are intrinsically linked to its biological activity [15,16,24]. To understand this relationship, four commercial extracts were selected to ensure a breadth of reported biological and chemical properties. This variation is not possible with fucoidans produced from the same seaweed species or extraction process. The selected extracts underwent a rigorous characterization using a variety of techniques; a summary of the analysis is provided in Table 1.

### 2.1. Structural Characteristics of Extracts

**Figure 1:**
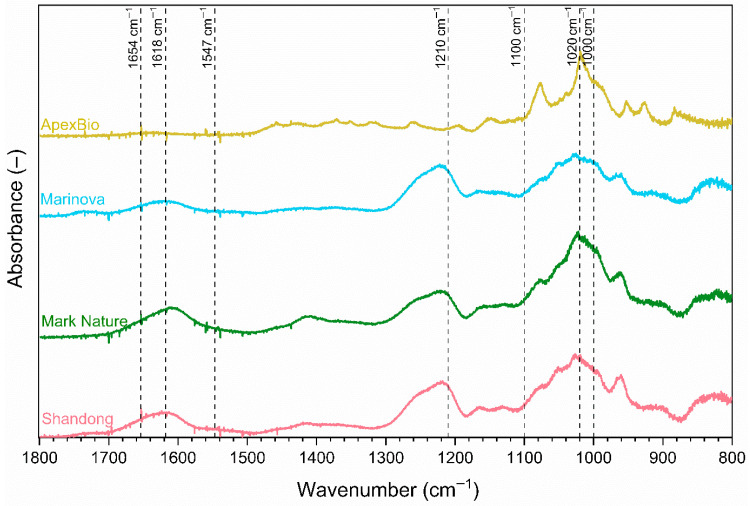
Analysis of the four commercial fucoidan extracts’ chemical structures by FTIR spectroscopy. Data collected using a resolution of 0.5 cm^−1^. Shandong (pink), Mark Nature (green), Marinova (blue) and ApexBio (yellow).

Notably, all the fucoidan extract’s IR spectra (Figure 1) contained bands between 1000 and 1100 cm^−1^, a region characteristic of polysaccharides [25], in particular a broad band at 1020 cm^−1^ attributed to ring vibrations found in sugars. The absence of bands at 1654 cm^−1^ and 1547 cm^−1^, characteristic of amide I and II modes [25,26] in a polypeptide chain, confirms no proteins in any of the samples. Shandong, Mark Nature and Marinova show other commonalities not found in ApexBio. In particular, a band at 1210 cm^−1^, specific to the sulfate group found in fucoidans [25,27,28,29] and a band at 1618 cm^−1^ characteristic of a carbonyl stretch in uronic acids are present in Shandong, Mark Nature and Marinova samples, very much in line with the FTIR spectra of other reported fucoidans. These bands are completely absent in ApexBio, indicating the absence of sulfated polysaccharides and uronic acids in this sample.

### 2.2. Chemical Characteristics of Extracts

#### 2.2.1. Monosaccharide and Uronic Acid Content of Extracts

High-Performance Anion-Exchange Chromatography with Pulsed Amperometric Detection (HPAEC-PAD) was used to determine monosaccharide and uronic acid content [16,30,31] (Data in absolute concentrations are provided in Supplementary Table S2).

Fucose is the primary monosaccharide of fucoidan [32,33], and therefore can be used as an indicator of extract purity. Marinova contained the highest amount of fucose, suggesting it is the purest sample, closely followed by the Shandong and Mark Nature extracts, with fucose completely absent in the ApexBio sample (Table 1).

**Table 1:** Commercial fucoidan extract (Shandong, Mark Nature, Marinova, ApexBio) chemical contents and characteristics. Monosaccharide and uronic acid content determined via HPAEC-PAD (*n* = 3). Molecular weight characteristics determined using SEC-MALS analysis (*n* = 1). Sulfation degree and protein content were determined using CHNS analysis (*n* = 3); in the case of ApexBio, sulfation degree was calculated using data from ICP-OES analysis (*n* = 3) and calculations set out by Zayed et al. [34]. Phenolic content was determined using the Folin–Ciocâlteu assay (*n* = 3). Data reported as mean ± SD. Different letters in the same row indicate a statistical difference between samples using a 95% confidence interval.

	Sample	Shandong	Mark Nature	Marinova	ApexBio
**Monosaccharide Content**	Fucose (%)	38.04 ± 2.40 ^a^	8.62 ± 2.29 ^b^	39.50 ± 5.88 ^a^	0.00 ± 0.00 ^b^
	Glucose (%)	5.80 ± 0.41 ^b^	69.00 ± 3.92 ^a^	2.20 ± 0.57 ^bc^	0.00 ± 0.00 ^c^
	Mannitol (%)	0.42 ± 0.00 ^b^	0.00 ± 0.00 ^b^	0.00 ± 0.02 ^b^	100.00 ± 2.60 ^a^
	Arabinose/Rhamnose (%)	2.80 ± 0.00 ^a^	0.46 ± 0.10 ^b^	0.00 ± 0.50 ^b^	0.00 ± 0.00 ^b^
**Uronic acid** **Content**	Glucuronic Acid (%)	24.08 ± 0.57 ^a^	2.21 ± 0.76 ^b^	3.63 ± 2.74 ^b^	0.00 ± 0.00 ^b^
	Guluronic Acid (%)	0.00 ± 0.15 ^b^	1.19 ± 0.31 ^a^	0.14 ± 0.00 ^b^	0.00 ± 0.00 ^b^
	Mannuronic Acid (%)	1.10 ± 0.46 ^c^	12.92 ± 2.14 ^a^	7.43 ± 0.57 ^b^	0.00 ± 0.00 ^c^
**Extract Properties**	Mn (kDa) ^†^	22.31 ± 0.08	2.30 ± 0.08	8.88 ± 0.16	1.61 ± 0.05
	Mw (kDa) ^‡^	49.46 ± 0.33	36.54 ± 0.25	24.91 ± 0.23	9.65 ± 0.09
	Polydispersity (−)	2.22 ± 0.02	15.87 ± 0.55	2.81 ± 0.06	6.00 ± 0.18
	Sulfation Degree (−)	0.86 ± 0.16 ^a^	0.13 ± 0.01 ^b^	0.88 ± 0.01 ^a^	0.01 ± 0.00 ^c,^*
**Extract** **Impurities**	Protein Content (%)	5.10 ± 0.42 ^a^	1.51 ± 0.06 ^c^	3.32 ± 0.03 ^b^	1.10 ± 0.03 ^c^
	Polyphenol Content (mg_GAE_ g_sample_^−1^) ^§^	0.70 ± 0.41 ^b^	0.36 ± 0.65 ^b^	7.11 ± 1.04 ^a^	1.68 ± 0.10 ^b^

* Sulfation degree calculated using sulfur content determined using ICP-OES, ^†^ Number average molecular weight, ^‡^ Weight average molecular weight, ^§^ Gallic acid equivalent.

The only monosaccharide detected in the ApexBio sample was mannitol, consistent with the observed FTIR spectra (Figure 1). Mannitol can be derived from laminarin or found free in brown seaweed [35], but is also present in numerous other natural sources, including fungi, bacteria, yeasts and plants [36]. So it is possible that this extract is not of seaweed origin.

Notably, no uronic acids were detected in ApexBio. However, both mannuronic (ManA) and guluronic (GulA) acids were detected in Mark Nature (ManA 12.92%, GulA 1.19%) and Marinova (ManA 7.43%, GulA 0.14%); these uronic acids derive from alginate [37], suggesting the presence of this polysaccharide in these extracts. Glucuronic acid was detected in the Shandong (24.08%), Mark Nature (2.21%) and Marinova (3.61%) extracts. This component derives from fucoidan, suggesting the presence of this polysaccharide in the extract.

#### 2.2.2. Molecular Weight of Extracts

The molecular weight of fucoidans is typically between 10 and 1000 kDa [16,17,18,19,20], with the size of the molecule dependent on environmental factors, e.g., seaweed species, harvest geolocation, season and seaweed maturity and process factors, i.e., the pre-treatment, extraction and purification methods selected and the conditions they are operated at [15].

The molecular weight characteristics of the four commercial extracts were studied using Size Exclusion Chromatography with Multi-angle Static Light Scattering (SEC-MALS) and refractive index (SEC-RI) detectors. These detectors were employed due to their ability to determine molar mass without the use of calibration standards [38] and its wide use in the characterization of fucoidans’ molecular size [38,39,40]. Table 1 presents the average molecular weights for the four commercial extracts (Full chromatograms in Supplementary S3.1), with all samples falling within the typical molecular weight range of fucoidans. Notably, ApexBio had the lowest molecular weight (<10 kDa), corresponding to fewer than 10 monosaccharides. The other three commercial samples showed molecular weights of medium size (24 to 50 kDa).

The polydispersity index (PDI) indicates the broadness of a molecular weight distribution; a value around 1 indicates a homogeneous sample with a narrow distribution, while a value greater than 1.5 indicates a dispersed sample, characteristic of natural polysaccharides. The commercial extracts had a PDI ranging from 2 to 16. The Mark Nature extract was the most polydisperse (15.87), suggesting this sample contains a range of macromolecules, including polysaccharides, proteins and other seaweed cell wall components.

#### 2.2.3. Elemental Content of Extracts

It is well established that a high sulfate content of fucoidan enhances its biological activity [4,33,41,42] and, as such, sulfur content was a potential key quality attribute to be explored. To determine sulfur content, CHNS elemental and ICP-OES analyses were employed (See Supplementary Table S1). The highest sulfation degree was found in the Marinova extract (0.88), followed by Shandong (0.86) and Mark Nature (0.13), with almost no sulfur (0.06%) detected in ApexBio, setting it apart from the rest. In terms of protein content, only a small amount was found in extracts (1–5%) and coupled with the lack of amine bands within the extracts’ FTIR spectra, this confirmed that the extracts contained negligible protein content.

#### 2.2.4. Phenolic Content of Extracts

A number of phenolic compounds are found in brown macroalgae, including a unique class called phlorotannins [43]. These compounds are used to protect seaweed from UV radiation and pathogens [43,44]. Polyphenols are coextracted with fucoidan and can bind to the molecule’s surface [45]. These are generally considered impurities in fucoidan extracts [31], but some argue that the presence of phenolics can enhance biological activity [46]. Therefore, it is important to determine the quantity of phenolics within a fucoidan sample by using the Folin–Ciocâlteu method.

The phenolic contents (Table 1) show that Marinova is relatively rich in polyphenols (56.49 ± 1.06 mg_GAE_ g_sample_^−1^), with Shandong (9.18 ± 1.11 mg_GAE_ g_sample_^−1^), ApexBio (8.73 ± 0.44 mg_GAE_ g_sample_^−1^) and Mark Nature (4.5 ± 0.14 mg_GAE_ g_sample_^−1^) having significantly lower total phenolic contents (>80%).

### 2.3. Summary of Extracts Chemical Content and Structural Characterization

Several interesting observations emerge from this detailed characterization. Shandong and Marinova were remarkably similar in sulfation degree (0.86 and 0.88) and fucose content (38.03% and 39.43%), but differed in molecular weight, with Shandong being a much larger molecule (22.31 kDa and 8.88 kDa). Marinova, on the other hand, contained significantly more phenolics. There is an ongoing debate over whether polyphenols enhance [46] or hinder [47] bioactivity. This detailed characterization provides a basis for testing this hypothesis.

The Mark Nature extract was mostly comprised of the neutral monosaccharide glucose and was less sulfated (0.13) than the Shandong and Marinova extracts. Additionally, this sample had the highest PDI, reflecting the variety of components, including monosaccharides, uronic acids, and phlorotannins, compared with the other extracts.

Throughout the analyses, the ApexBio extract emerged as an outlier compared to the three other commercial extracts. ApexBio’s FTIR spectra (Figure 1) were distinct, lacking the characteristic bands of sulfated fucoidan and uronic acids, with bands characteristic of mannitol present. Furthermore, the monosaccharide and uronic acid profile, obtained under the same analytical conditions as the other samples, revealed that only mannitol was present in the ApexBio, with fucose, the primary monosaccharide of fucoidan, completely absent. Therefore, the ApexBio sample cannot be classified as a fucoidan, illustrating a common issue in natural product research: mislabeled extracts due to a lack of standardization and regulation. As such, the ApexBio sample was excluded from subsequent biological testing.

### 2.4. Biological Activity Assessment of Extracts

#### 2.4.1. Antioxidant Activity

**Figure 2:**
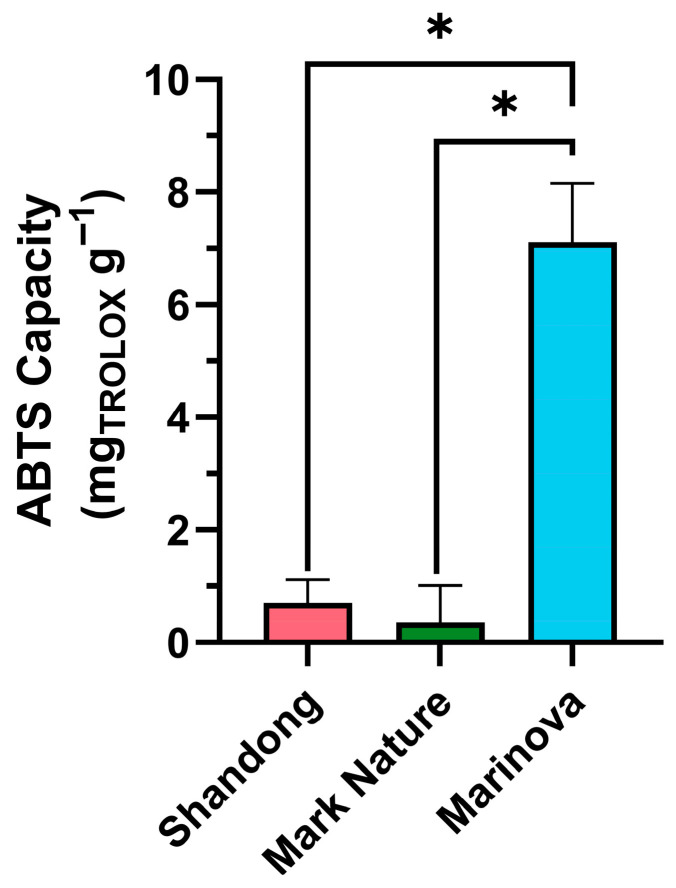
Antioxidant capacity of fucoidan extracts determined by ABTS assay. Error bars represent mean ± standard deviation (SD), *n* = 3. Statistical data were analyzed using a one-way ANOVA with post hoc Tukey test (* *p* < 0.05, between groups) using Prism 10 (GraphPad).

The three fucoidans (Shandong, Mark Nature, Marinova) exhibited some degree of antioxidant activity (Figure 2). The Marinova extract’s activity was significantly the highest, followed by Shandong, with Mark Nature possessing the lowest activity. This trend qualitatively matches the polyphenol contents in the samples, in line with Obluchinskaya et al. who found the antioxidant contents of fucoidan extracts correlated strongly with their phenolic content [48]. While seaweed polyphenolic compounds possess antioxidant properties [49], there is conflicting evidence in the literature regarding whether the presence of phenolic compounds in fucoidan extracts hinders or enhances antioxidant activity [47].

#### 2.4.2. Antiviral Activity

As previously reported, fucoidan has displayed antiviral activity against many viruses, including coronavirus [50], human immunodeficiency virus [51] and multiple members of the Paramyxoviridae family, such as Newcastle disease [52] and parainfluenza types 1 [53] and 2 [54].

This study focused on Nipah virus, another member of the Paramyxoviridae family. This virus is an emerging zoonotic infectious disease [55] identified as a priority for research and development by the World Health Organization (WHO) [56,57] due to its high mortality rates of 80.1% [58] and the large number of people (176.2 million) living in endemic risk areas [57]. Nipah virus is heavily reliant on cellular polysaccharides for initial cell interaction [59], suggesting the potential for competitive inhibition of the virus by the introduction of other polysaccharides, as seen with heparan sulfate [60,61]. To study Nipah virus safely, a lentiviral-based pseudotype was used [62,63]. Pseudotypes are viral particles composed of the core of one virus decorated with glycoproteins from another virus and incapable of completing a full viral replication cycle [64,65].

**Figure 3:**
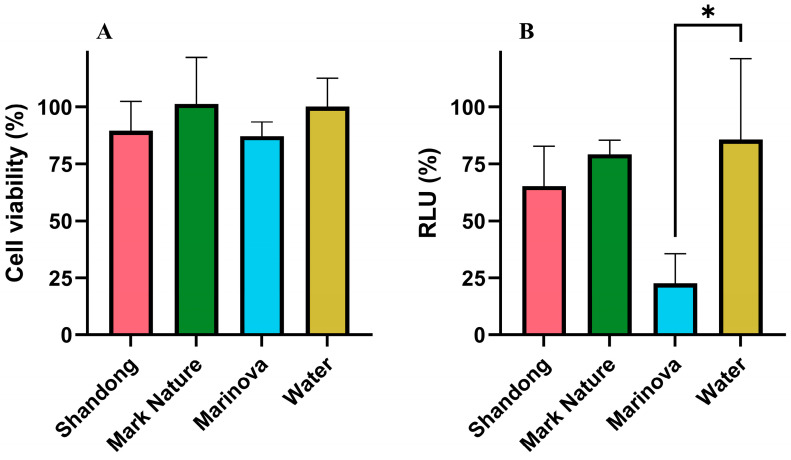
Fucoidans’ effect on cell viability and virus transduction (**A**). Cell viability of HEK293T cells after exposure to commercial fucoidans (1 mg mL^−1^). Cell metabolic activity was assessed by resazurin assay 48 h post-transduction. Data points were normalized to the negative control (no virus or fucoidan treatment) to calculate cell viability. (**B**). HEK293T cells were transduced with Nipah lentivirus pseudotypes in the presence of commercial fucoidan extracts (1 mg mL^−1^) or water for four hours. Cells were lysed and viral presence analyzed by luciferase assay 48 h post-transduction. Data points were normalized to cell viability (determined by resazurin assay) and then the positive control (no virus treatment), to give a relative luminescence unit (RLU) reading. Error bars represent mean ± standard deviation (SD), *n* = 3 (3 biological replicates with 3 technical replicates). Statistical data were analyzed using a one-way ANOVA with post hoc Tukey test (* *p* < 0.05, vs. water control) using Prism 10 (GraphPad).

A common risk with antiviral therapeutics is that they might affect host cell function. However, no cytotoxic effect on the HEK293T cells was observed for any of the three fucoidan extracts, with no difference between groups and the water control, suggesting that the cells remained viable in the presence of all fucoidan extracts (Figure 3A). All extracts appeared to reduce the amount of virus present within the cells, with a clear reduction observed for the Marinova sample compared to the water control, suggesting that this sample was a potent antiviral against the Nipah pseudotype (Figure 3B).

The lentiviral-based Nipah pseudotypes are incapable of completing the full viral replication cycle and exiting the host cell, i.e. attach and enter the cell before the luciferase gene is expressed by infected cells. As has been shown in the literature, other polysaccharides, such as heparan sulfate and dextran sulfate [60,61], inhibit these early stages of viral infection [66]. This study suggests that fucoidan acts similarly, inhibiting the attachment and entry phases of the viral cycle, complementing the literature’s view that it affects the early stages of the viral replication cycle, most likely from interactions between the fucoidan molecule and viral surface glycoproteins, thereby interfering with the virus’s ability to attach to and enter cells [5]. Nosik et al. demonstrated that fucoidan inhibits the attachment and entry of the viral replication process by varying the time of addition of fucoidan treatment when studying HIV-1 virus [6]. Thuy et al. observed that viral inhibition occurred if HIV-1 was preincubated with the fucoidan [7], further suggesting that the molecule likely affects the attachment and entry stages of viral replication. Yan et al. found that fucoidan affected the entry of these viruses into the host cell by targeting its spike protein and inhibiting the cell receptor protease furin [67], typically responsible for the cleavage of the coronavirus spike protein during the entry phase [68]. Kwon et al., through correlation of modeling with antiviral activity, found that the greater the binding efficiency of a polysaccharide to the SARS-CoV-2 spike protein, the greater the antiviral effect of the fucoidan [69]. Therefore, our results align with the published evidence, and the consensus view is that fucoidan prevents viral infection through inhibition of the attachment and entry replication phases across a broad spectrum of viruses, including Nipah virus.

Further investigations are required to confirm this hypothesis. An antibody-based attachment and entry assay could measure the amount of p24 protein on and within the cell arising from the HIV-1 lentivirus capsid, indicating fucoidan’s mechanism of action [70]. Another virus not based on the lentivirus system should also be tested to rule out the small possibility that fucoidan inhibits the HIV-1 core. These studies would then enable the characterization of the specific receptors and glycoproteins affected by fucoidan.

#### 2.4.3. Antifungal Activity

The emergence of antifungal-resistant organisms is a critical issue with fungal infections currently responsible for 3.8 million global deaths annually [71]. Treatment is reliant on four main classes of antifungal drugs: azoles, echinocandins, polyenes and flucytosines [72], highlighting the need for discovery of new antifungal treatments. A fungal pathogen with devastating effects is *Cryptococcus neoformans*, which is responsible for the deaths of 223,100 HIV/AIDS patients per year [73,74]. Furthermore, *Candida* species are amongst the most common human fungal pathogens [75], responsible for between 250,000 and 700,000 cases of hospital-acquired invasive candidiasis per year, with mortality rates ranging from 40 to 55% [76]. Accordingly, *C. neoformans*, *C. auris*, and *C. dubliensis* were selected for this study.

Prior antifungal studies have used relatively high fucoidan concentrations to assess whether any activity is present. Oka et al. found a statistically significant fungal inhibition at 100 mg/mL [77]. For fucoidans to be used in biomedical therapeutics, this concentration needs to be greatly reduced. As such, testing was carried out at a modest 5 mg/mL, thereby respecting the solubility limits imposed by the viscosity of concentrated fucoidan solutions.

**Figure 4:**
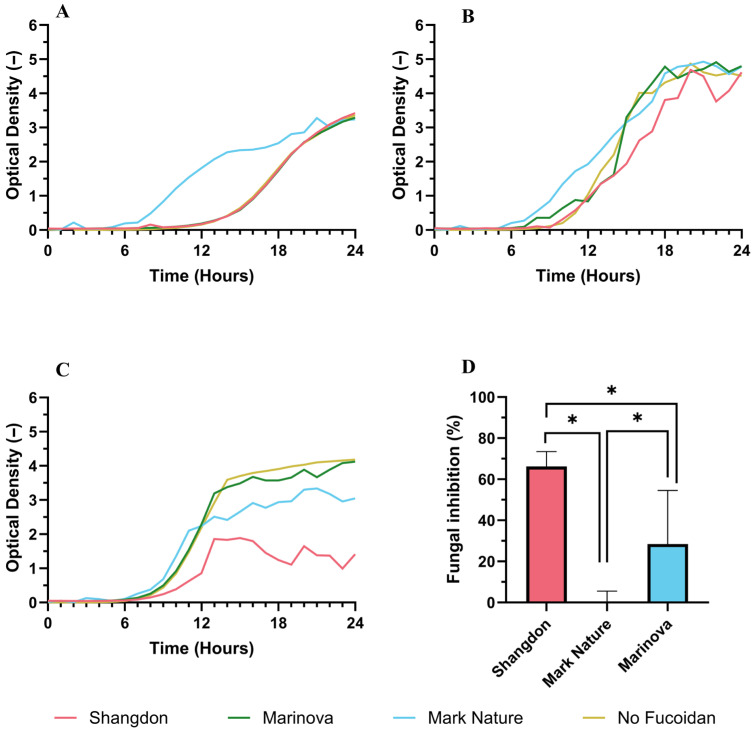
Effects of fucoidan on fungus growth. Growth curve studies showing inhibitory effects of fucoidans (5 mg mL^−1^) on (**A**) *C. neoformans*, (**B**) *C. auris*, and (**C**) *C. dubliensis* were determined by hourly measurements of optical density (600 nm) over 24 h. (**D**) Fungal inhibition in *C. dubliensis* growth vs no fucoidan control. Growth curves plotted show mean OD600 values using three biological replicates. Error bars represent mean ± standard deviation (SD), *n* = 3 (3 biological replicates with 3 technical replicates). Statistical data were analyzed using a one-way ANOVA with post hoc Tukey test (* *p* < 0.05, between groups) using Prism 10 (GraphPad).

A slight pro-fungal phenotype was observed with the Mark Nature extract against *C. neoformans* (Figure 4A), which may be explained by the extract’s high glucose content serving as a carbon source for the fungus. No antifungal phenotype was observed for any of the samples in *C. auris* (Figure 4B). A noticeable antifungal effect was seen in *C. dubliensis,* with Shandong (66%) and Marinova (28%) significantly inhibiting the growth of this fungal strain (Figure 4C,D).

Surprisingly, despite the close phylogenetic relationship between *C. dubliensis* and the more prevalent *Candida albicans*, no inhibition of the latter was observed (See Supplementary Figure S1). Therefore, further investigation is required to determine the cause of this antifungal specificity, and future development may focus on formulating fucoidan to enable lower, more medically applicable dosages.

#### 2.4.4. Antibacterial Activity

It has been well documented that antibiotic resistance is on the rise due to the heavy reliance on and misuse of existing antibiotics [78]. In 2019, resistant bacteria were associated with 4.95 million deaths [79]. Given the lack of new antibacterial drugs [78], natural products such as fucoidan present an opportunity. *Klebsiella pneumoniae* was selected for this study due to the clear threat it presents as the leading cause of highly multidrug-resistant, healthcare-acquired infections [80].

**Figure 5:**
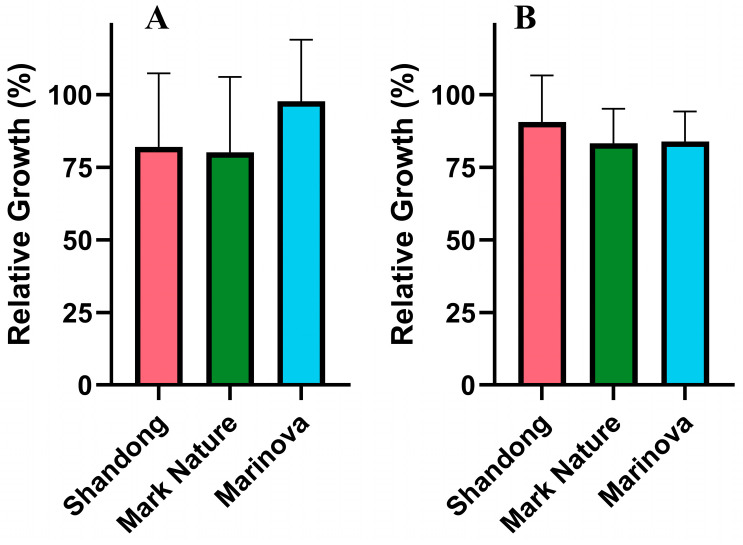
Antibacterial effect of commercial fucoidans extracts on *K. pneumoniae* at a concentration of (**A**) 2 mg/mL and (**B**) 5 mg/mL determined by ‘polysaccharide survival assay’. Error bars represent mean ± standard deviation (SD), *n* = 3 (3 biological replicates with 3 technical replicates). Statistical data were analyzed using a one-way ANOVA with post hoc Tukey test (* *p* < 0.05, between groups) using Prism 10 (GraphPad).

No clear antibacterial effect was observed for any of the extracts tested at either 5 or 2 mg/mL against *K. pneumoniae*, with all relative growth to the control being around 100% (Figure 5). Testing at a higher dose with a longer incubation time warrants exploration in future research.

Ayrapetyan et al. also worked with *K. pneumoniae* at higher concentrations than this study (16 and 24 mg/mL), observing a mild inhibition of growth (~60%) in the presence of crude and membrane-purified fucoidans, with the other bacterial strains tested experiencing strong, near-complete (>90%) growth inhibition. The purified sample was a less potent antibacterial [12], likely due to the removal of low molecular weight components during the membrane filtration process.

Although not observed in this study, fucoidan has shown antibacterial activity against other bacterial species. Beagan et al. observed inhibition of *Staphylococcus aureus*, *Streptococcus mutans* and *Streptococcus sanguinis* growth with fucoidan minimal inhibitory concentration (MIC) ranging from 3 to 24 mg mL^−1^, depending on the fucoidan fraction tested. Alboofetileh et al. observed inhibition of the growth of *Pseudomonas aeruginosa* and *Escherichia coli*. depending on the fucoidan extraction method used [13].

McGurrin et al. suggested that the bacterial growth rate and lag parameters were correlated directly to fucoidan structure and contents, observing that bacterial lag increased in the presence of the fucose [23]. Another possibly important quality attribute is molecular weight. Cabral et al. found that of several different molecular weight samples tested, the largest molecular weight fraction (>300 kDa) had a statistically significant longer lag time of *Listeria innocua*, suggesting this may be an important parameter. Still, it remains unclear if this was due to a difference in composition or molecular weight [17].

Further work should now focus on understanding what key quality attributes a fucoidan must possess to have antibacterial activity; this may be aided by mechanistic studies aimed at understanding how the fucoidan molecule interacts with different bacterial strains and how attributes such as molecular weight and sulphation degree play a role.

#### 2.4.5. Prebiotic Activity

Probiotic bacteria are a diverse group of microbes that, when present at sufficient amounts in the human body, confer a health benefit [81,82], including prevention of disease [83] and inflammation [84]. When prebiotic substances are utilized by probiotic microorganisms, their activity or functionality is enhanced [85]. Fucoidan is considered to be a prebiotic due to its ability to enhance the growth [22,86,87] and short-chain fatty acid production [88] of probiotic bacteria. In this study, *Lactobacillus casei* was selected for its widespread commercial use in prebiotic supplements [89] and dairy products [90]. The reported health benefits of *L. casei* include modulation of the gut microbiome [91], enhanced immune response following vaccinations [92] and greater resistance to infection [93].

**Figure 6:**
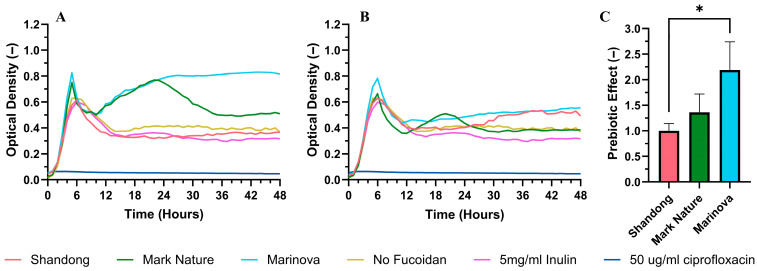
Effects of fucoidan on *L. casei* growth. Growth curve studies showing inhibitory effects of fucoidans at a concentration of (**A**) 5 mg/mL and (**B**) 2 mg/mL determined by hourly measurements of optical density (600 nm) over a 48 h period. (**C**) Prebiotic effect is defined as relative *L. casei* growth with respect to the no fucoidan control achieved at the stationary growth phase (~20 h). Growth curves plotted show mean OD600 values using three biological replicates. Error bars represent mean ± standard deviation (SD), *n* = 3 (3 biological replicates with 3 technical replicates). Statistical data were analyzed using a one-way ANOVA with post hoc Tukey test (* *p* < 0.05, between groups) using Prism 10 (GraphPad).

A prebiotic effect (enhanced growth of probiotic bacteria) was observed for *L. casei* in the presence of two fucoidans (Mark Nature and Marinova), as evidenced by increased optical density compared to the no-fucoidan control at a concentration of 5 mg/mL (Figure 6A). The strength of this effect was greatly reduced at 2 mg/mL (Figure 6B), with no effect present at lower fucoidan concentrations tested (See Supplementary Figure S2).

A spike in the initial optical density reading was observed, explained by the extract mixing into the growth broth. Both Marinova and Mark Nature demonstrated sustained prebiotic effects, as indicated by prebiotic effect scores of 2.19 and 1.36, respectively, with Marinova being the more potent prebiotic among the commercial samples. This score is limited because cultures enter their death phase at different rates, and as such, defining an accurate prebiotic effect remains a challenge.

Nevertheless, this work confirms that fucoidan enhances the growth of *L. casei*. Habibi et al. found that fucoidan enhanced the growth of other *Lactobacillus* species (*L. acidophilus*, *L. plantarum*, *L. gasseri*, *L. paracasei*, *L. reuteri*, *L. rhamnosus GG*) [86]. While Okolie et al. observed an increase in the growth rate of *L. delbruecki* subsp. *bulgaricus* [22]. In vivo, Yang et al. observed an increase in the abundance of several beneficial gut bacteria, including *Lactobacillus*, when mice were fed diets supplemented with fucoidan [94].

## 3. Discussion

The resulting chemical structure and characteristics of a fucoidan are dependent on both environmental and processing factors, making consistent production of a biologically active fucoidan challenging. Furthermore, the specific quality attributes an extract requires for a given bioactivity remain unclear. This study builds upon McGurrin et al. [23] by applying a formalized correlation approach to identify potential attributes that enhance antioxidant, antiviral, antifungal, and prebiotic activities for further exploration.

### 3.1. Bioactivity Correlation Matrix

**Figure 7:**
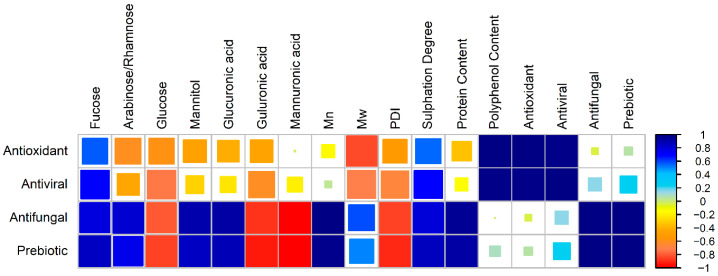
Pearson’s correlation matrix of antioxidant, antiviral, antifungal and prebiotic biological activities with fucoidan extract composition, molecular weight and bioactivity. The size of the square indicates the strength of correlation, with color indicating positive or negative correlations. Data was analyzed using the ‘corrplot’ function in RStudio version 4.1.1.

To explore any potential links between the chemical and structural properties of the investigated fucoidans and their biological activity, a Pearson’s correlation matrix was created (Figure 7). We note here that only the Shandong, Mark Nature, and Marinova samples were considered, as ApexBio lacks many of the typical fucoidan characteristics. Also, the antibacterial results were excluded from the correlation analysis as no inhibitory effect was observed with any of the tested samples. Given the limited sample size (*n* = 3) used in this analysis, the identified correlations should only be interpreted as exploratory, requiring further verification.

Notably, the four biological activities (antioxidant, antiviral, antifungal and prebiotic) were positively correlated with fucose, the primary monosaccharide in fucoidan [32,33]. This suggests that the observed biological activities arise directly from the presence of fucoidan, as has been widely reported in the literature [2,3,4,5,6,7,8,9].

Furthermore, a negative correlation was suggested between antioxidant and antiviral activities with the presence of other monosaccharides, e.g., glucose and mannitol, further suggesting that biological activity is improved by the purity (fucose content) of fucoidan extracts. Interestingly, the presence of glucose was negatively correlated with antifungal activity, which may be explained by *Candida* using glucose as a nutrient source and rapidly consuming the sugar [95]. A similar trend should be observed with *L. casei*, which also consumes glucose during its growth [96], but, instead, prebiotic activity decreased with the presence of glucose; this difference requires further investigation.

Within the literature, there is debate on whether small or large molecular weights enhance biological activity. Interestingly, the correlation analysis suggests that this discrepancy is dependent on the bioactivity tested. The correlation analysis suggested that antioxidant activity is improved by smaller molecular weights, aligning with the literature’s consensus view. Chen et al. found that the antioxidant activity of fucoidans increased after digestion, reducing the extract’s molar mass [4]. Similarly, Geun Lee et al. observed a reduction in reactive oxygen species in zebrafish, with the antioxidant effect increasing with the reduction in molecular weight of fractions [97]. It has been suggested that the high viscosity of large molecular weight samples prevents fucoidan’s ability to permeate into cells or through a liquid medium to scavenge free radicals [98].

Similarly, this work suggests that antiviral activity increases as molecular weight is reduced. Dinesh et al. found a 30% greater inhibition of reverse transcriptase activity and the p24 antigen of HIV-1 when using a lower molecular weight fraction compared to the crude sample [51]. Similarly, Krylova et al. observed a lower IC_50_ value when an enzymatically modified lower molecular weight extract (~50 mg/mL) fraction was used instead of a larger crude fucoidan (~100 mg/mL) [99], suggesting viral inhibition increased as molecular weight decreased. Conversely, Sun et al. observed a ~10% decrease in SARS-CoV-2 inhibition with reducing molecular weight at a fucoidan concentration of 12.5 µg/mL [100]. This conflict in the literature can be divided into two arguments: some studies suggest that high molecular weight molecules can more effectively block viral binding to host cell receptors [100], while others argue that low molecular weight extracts enhance bioavailability [101,102]. Further work is required to determine the underlying mechanisms of fucoidan as an antiviral agent, so the source of debate can be tested.

Furthermore, this analysis suggests that prebiotic activities are increased by the presence of large molecular weight, low polydispersity fucoidan extracts. This is in contrast to the work of Sun et al., who suggested that fucoidans with a lower molecular weight had a greater effect on *Lactobacillus* growth [103]. This difference likely arises from differences in the study’s scope, with an entire microbial family being considered rather than this study’s approach, which focused on a specific species, highlighting the need to consider which fucoidan quality attributes are necessary for each application on a case-by-case basis. The correlation analysis also suggests that antifungal activities are improved by large polysaccharides with little branching. There is a gap regarding this area, with few studies examining the effect of molecular weight on this activity.

Notably, all bioactivities tested were positively correlated to sulfate content; it is well documented in the literature that maximizing this key quality improves biological activity [4,5,104,105]. Chen et al. found a positive correlation between antioxidant content and sulfate content [4]. It has been suggested that the increased sulfate content increases the ability of the molecule to scavenge free radicals [2,106].

In terms of antiviral activity, Mandal et al. observed a reduction in antiviral potency of desulfated samples in comparison to sulfated samples [104]. It has also been suggested that for antiviral activity, the sulfate group location is important, with HSV-2 inhibition dependent on a sulfate group being present on the C4 of the (1–3)-linked fucopyranosyl unit [5,104,105]. It is theorized that the negative charge of the sulfate groups interacts with the viruses’ positively charged surface glycoproteins, disrupting their ability to attach to the cell surface [5,107]. However, there remains a large knowledge gap on the underlying mechanisms of antifungal and prebiotic activities and the role sulfate plays in this bioactivity.

Finally, there is a debate as to whether polyphenols should be considered as impurities in fucoidan extracts [31], and as to whether polyphenols enhance [46] or hinder bioactivity [47]. This analysis suggests once again that the presence of phenolics’ effect is dependent on the specific bioactivity to which the extract is applied, but requires further exploration.

### 3.2. Key Limitations of This Study

There are some notable limitations that must be recognized when interpreting these results. The main limitation of this study is that results from the correlation analysis should only be interpreted as preliminary, requiring further experimentation and validation. This limitation arises from the small sample size (*n* = 3) used in the correlation analysis, meaning the correlations cannot be interpreted as causal or robust. However, this work does provide a formalized method for identifying possible key characteristics of fucoidan that merit further investigation.

Additionally, while the commercial samples used in the study provided a breadth of reported biological and chemical properties, they introduced several uncontrolled variables. Each fucoidan is derived from a different seaweed species, harvested from various geolocations and seasons, and produced using a proprietary production process. These factors should be considered when discussing the correlations identified.

Finally, each measured bioactivity was determined using specific microorganisms, which may not apply to a wider microorganism family. For example, the phenotypic effects of fucoidan observed against *C. dubliensis* did not extend to other fungal species.

### 3.3. Future Work

It is often stated that correlation does not equal causation. Therefore, the correlation matrix requires further validation. In addition, the limited sample size means that the results in this study can only be interpreted as hypothesis-generating. As such, further experimentation is required.

To build on this work and further develop confidence in the correlations identified in this study, the formalized approach should be expanded to a larger dataset to include more fucoidan extracts that have undergone detailed chemical characterization. This should include the study to further microbial species not tested in this study, for example, in the prebiotic work, the effects of fucoidan on *Bifidobacterium*, *Lactococcus*, *Bacillus* and *Streptococcus* growth should be examined. With high-throughput screening assays using liquid handling systems, enabling the testing of a large number of samples against many microbes [108].

To verify the sulfate–function correlation, fucoidan extracts should be produced before undergoing an anion exchange (AEX) chromatography process to produce highly sulfated polysaccharides [109]. Similarly, fucoidans of various molecular weights could be produced by a membrane [17,110] or size exclusion chromatography [111,112,113] fractionation process. These naturally occurring fucoidans could then be employed in experimental assays to quantify the potency of extracts before and after modification of sulfate content or molecular weight. After which, mechanistic studies should use knockout bacteria [114] or, for antiviral activity, an antibody-based attachment and entry assay measuring the amount of virus protein on and within the cell could be used [70]. These proposed assays would aid in the determination of how the fucoidan sulfate group interacts with the microorganism and the effect of molecular weight on bioavailability.

## 4. Materials and Methods

### 4.1. Selection of Commercial Fucoidans

This study focused on commercially available fucoidan extracts to enable replication of tests used here and to allow further exploration of the bioactivities presented with alternative assays. This is not currently possible with extracted fucoidans without the exact seaweed biomass and identical conditions. Fucoidan extracts were selected to represent those available on the market and to ensure variation in the chemical composition of fucoidans. Four commercial products were purchased from Shandong (Shandong Jiejing Group, Rizhao, China), Mark Nature (MarkNature, Fullerton, CA, USA), Marinova (Marinova Pty Ltd., Cambridge, Australia), and ApexBio (APExBIO Technology LLC, Houston, TX, USA).

Fucoidan extracts were selected to represent those available on the market, considering several factors. Initially, inquiries were sent to nine producers, with two excluded after failing to respond or no longer producing fucoidan. The remaining seven were narrowed down to four for chemical characterization and bioactivity assessments using several considerations. Each selected fucoidan extract was produced using a proprietary extraction method, ensuring diversity of processing techniques. The next criterion was seaweed species, as it has been demonstrated that the chemical profile of a fucoidan is heavily dependent on the raw material used [33,115]. Extracts were selected to represent common brown seaweed species, with Shandong and Mark Nature derived from *Laminaria japonica*, Marinova from *Undaria pinnatifida*, with the origin of ApexBio being unknown. Geographic location has also been demonstrated to affect fucoidan extract characteristics depending on many factors (e.g., sea temperatures and tidal patterns) [15,116], with Shandong originating from China, Mark Nature from the United States, Marinova from Australia and ApexBio’s origin unknown. Finally, the extracts selected were reported to possess many biological activities, including anticancer, anticoagulant, prebiotic, antioxidant, antiviral, neuroprotective, and immune-modulating effects [117,118,119,120].

### 4.2. Chemical and Structural Characterization of Fucoidan Extracts

#### 4.2.1. Fourier Transform Infrared Spectroscopy

Fourier-transform infrared (FTIR) spectroscopy has been widely applied to examine fucoidan extracts’ structure [29,121,122,123]. This technique was used for the identification of the functional groups present within fucoidan samples. FTIR spectra were obtained using a Nicolet iS10 spectrometer equipped with a Smart iTX diamond attenuated total reflector (Thermo Fisher Scientific, Waltham, MA, USA) covering the wave range of 400–4000 cm^−1^ with a resolution of 0.5 cm^−1^ with 16 scans utilizing OMIC Spectra software (version 9.8, Thermo Fisher Scientific, Waltham, MA, USA).

#### 4.2.2. Size Exclusion Chromatography with Multi-Angled Light Scattering Detector (SEC-MALS)

The molecular weight profile of the four fucoidan samples was analyzed using Size Exclusion Chromatography with Multi-Angled Light Scattering Detector (SEC-MALS) as described in Apostolova et al. (2022) [124]. Fucoidans were analyzed on three size-exclusion columns in series: SB-806 HQ, SB-804 HQ, and SB-803 HQ, 300 mm L × 8 mm I.D., Shodex (Resonac, Tokyo, Japan) and detected on a MiniDAWN TREOS II multi-angle light scattering detector (Wyatt Technology, Goleta, CA, USA) and a RID-10A refractive index detector (Shimadzu, Kyoto, Japan) using a dn/dc value of 0.15 mL g^−1^.

#### 4.2.3. Elemental Analysis (CHNS)

The carbon, hydrogen, nitrogen and sulfur content of commercial samples was determined using elemental analysis. Commercial samples were dried at 50 °C for an hour to remove excess water content, before 2.5 mg of dried sample was added to 9 mg of vanadium pentoxide (Elemtex, Gunnislake, UK) in a tin capsule (Elemtex, Gunnislake, UK) before being compressed and sealed. Also prepared were reference standards of sulfanilamide (Elemtex, Gunnislake, UK), atropine (Elemtex, Gunnislake, UK) and a four-point calibration curve of 2,5-(Bis(5-tert-butyl-2-benzo-oxazol-2-yl) thiophene (BBOT) (Elemtex, Gunnislake, UK). After preparation, the samples and standards were stored in a desiccator.

Samples were subsequently analyzed on a Flash SMART 2000 instrument (Thermo Fisher Scientific, Waltham, MA, USA) equipped with a CHNS Prepacked Quartz Reaction Tube (Elemtex) with a 2 m PTFE CHNS Separation Column (CE Instruments, Wigan, UK) and a Thermal Conductivity Detector. Sulfation degree and protein content were calculated as described in Zayed et al. [34].

#### 4.2.4. Inductively Coupled Plasma-Optical Emission Spectrometry (ICP-OES)

Major and trace elements in the commercial samples were quantified using a Varian Vista Pro Inductively Coupled Plasma-Optical Emission Spectrometer (Agilent Technologies, Santa Clara, CA, USA). Approximately 100 mg of fucoidan was digested in ARISTAR^®^ grade nitric acid (VWR) before being washed and filtered. Reference standards of BHVO-1 Hawaiian Basalt (United States Geological Survey, Reston, VA, USA) and Fiji water were prepared with all samples stored at 4 °C prior to analysis.

#### 4.2.5. High Performance Anion Exchange Chromatography—Pulsed Amperometric Detection (HPAEC-PAD)

The monosaccharide and uronic acid content of the extracts was determined using an ICS 3000 Dionex HPAEC (Thermo Fisher Scientific, Waltham, MA, USA) coupled to a Pulsed Amperometric Detector (PAD with gold electrode). 10 mg of extract was hydrolyzed in 2 M trifluoroacetic acid (TFA) at 120 °C for 2 h before being dried using a miVac DUO Concentrator Speed Vac (GeneVac, Ipswich, UK) and stored at −20 °C. Before analysis, the hydrolyzed samples were dissolved in ultrapure water and filtered before being degassed.

The HPAEC-PAD system was pressurized with argon, and analysis was performed on two columns in series: 3 × 150 mm CarboPac PA20 Analytical Column (Thermo Fisher Scientific, Waltham, MA, USA) and 3 × 30 mm CarboPac PA20 Guard Column (Thermo Fisher Scientific, Waltham, MA, USA).

#### 4.2.6. Folin–Ciocâlteu Colorimetric Assay

The polyphenol content of extracts was determined through the Folin–Ciocâlteu colorimetric assay using a protocol adapted from de Falco et al. (2018) [125]. To a 96-well plate, 5.4 µL of sample dissolved in ultrapure water (10 mg/mL) was added alongside 108 µL of diluted 1:10 Folin–Ciocalteu reagent (Sigma-Aldrich, St. Louis, MO, USA) and 86.4 µL of 7.5% sodium carbonate (Thermo Fisher Scientific, Waltham, MA, USA) solution. An eight-point gallic acid (Sigma-Aldrich, St. Louis, MO, USA) standard curve was prepared and also included in the plate.

The 96-well plate was incubated at 50 °C for 5 min, and the absorbance at 760 nm of each well was determined using a CLARIOStar plate reader (BMG Labtech, Ortenberg, Germany). Phenolic content of samples was expressed as mg of gallic acid equivalent per gram of extract using a gallic acid standard curve. Samples were measured in triplicate, and standards were measured six times.

### 4.3. Biological Activity Assessment of Extracts

#### 4.3.1. Antioxidant Activity

The antioxidant capacity of extracts was assessed by 2,2′-azino-bis-(3-ethylbenzothiazoline-6-sulfonic) acid (ABTS) assay using a modified protocol from Falco et al. (2018), [125]. Briefly, an ABTS free radical (ABTS•) was prepared by the addition of 140 mM potassium persulfate (Sigma-Aldrich, St. Louis, MO, USA) solution to a 7 mM ABTS solution (Sigma-Aldrich, St. Louis, MO, USA) using a ratio of 88 µL per 5 mL of ABTS solution. The free radical (ABTS•) solution was stored at 4 °C, protected from light sources, overnight.

The ABTS• solution was diluted in deionized water to an absorbance at 734 nm of 0.700–750. An 8-point calibration curve of 6-hydroxy-2,5,7,8-tetramethylchroman-2-carboxylic acid (TROLOX) (Sigma-Aldrich, St. Louis, MO, USA) was prepared. 1 mL of the diluted ABTS• solution was added to 100 µL dissolved samples (5 mg/mL) or TROLOX standard solutions, and the absorbance at 734 nm was determined after 2 min and 30 s by NanoDrop^TM^ 2000 (Thermo Fisher Scientific, Waltham, MA, USA). The inhibition of the radical was calculated by subtracting from 1 the ratio of absorbance of the sample to the solvent blank. A standard curve was then plotted, and the antioxidant capacity of samples was expressed as mg of TROLOX equivalent per gram of extract.

#### 4.3.2. Antiviral Activity

HEK293T cells (ATCC CRL-3216) were cultured in Dulbecco’s Modified Eagle Medium (DMEM) supplemented with GlutaMAX (Gibco, Thermo Fisher Scientific, Waltham, MA, USA), 10% fetal bovine serum (Gibco, Thermo Fisher Scientific, Waltham, MA, USA), 1% penicillin-streptomycin (Gibco, Thermo Fisher Scientific, Waltham, MA, USA) and 2% Normocin (InvivoGen, San Diego, CA, USA) using an incubator set to 37 °C and 5% CO_2_.

Nipah lentivirus pseudotypes were prepared as described by Mak et al. (2024) [62]. A monolayer of HEK293T was seeded into a Petri dish at a cell density of 6.25 × 10^4^ cells/cm^2^ prior to incubation for 24 h. The cells were then transfected with four plasmids: p8.19 (GenScript Biotech, Nanjing, China) responsible for expressing the HIV-1 Gag, Pol and Rev lentivirus packaging, pCSFLW (Provided by Nigel Temperton, University of Kent, Canterbury, UK) encoding the firefly luciferase reporter gene and two pcDNA3.1 encoding the Nipah glycoprotein F (Provided by Ed Wright, University of Sussex, Brighton and Hove, UK) and glycoprotein G (Provided by Ed Wright, University of Sussex). To ensure uptake of this exogenous DNA through endocytosis [126], the transfection agent polyethylenimine (Polysciences) was included. The transfected cells were incubated for 72 h, after which the pseudotype was harvested by filtering the supernatant with 0.45 µm filters. The pseudotypes were stored at −80 °C to prevent degradation.

The antiviral activity of the four commercial fucoidan extracts was then assessed by a lentiviral pseudotype assay. A 96-well plate was seeded with 100 µL of supplemented DMEM containing HEK293T cells at a density of 9.1 × 10^4^ cells/cm^2^ and incubated overnight to allow a cell monolayer to form. The following day, cells were pre-treated with fucoidan by replacing the cell media with 100 µL of supplemented DMEM containing 1 mg/mL of each commercial fucoidan and the plate was further incubated for 24 h. Cells were transduced with the Nipah virus pseudotype by replacing the cell media in each well with 50 µL of unsupplemented DMEM containing 1 mg/mL of each of the commercial fucoidans and Nipah viral pseudotype at a concentration that would provide a luminescence signal of twenty in the negative control relative to the virus blank. The plate was returned to the incubator for four hours, and afterward, the inoculum was removed, and cells were post-treated with 100 µL of the same media used for the pre-treatment stage. The plate was then returned to the incubator for 48 h.

The viability of the HEK293T cells at the end of the transduction assay was determined using a resazurin assay, commonly used due to its high sensitivity [127]. The resazurin assay is a REDOX reaction in which metabolically active (viable) cells reduce resazurin (a blue low fluorescence molecule) to resorufin (a pink high fluorescence molecule) through oxidation of the coenzyme NADH to NAD+ [128].

To each well in the 96-well plate, 11 µL of 10X resazurin (Thermo Fisher Scientific, Waltham, MA, USA) solution was added before incubation at 37 °C for 2 h. The resulting plate was inspected to ensure that viable cells were present. The fluorescence of each well was measured on a CLARIOStar plate reader (BMG Labtech, Ortenberg, Germany). using an excitation of 570 nm and an emission of 590 nm. Cell viability was calculated by the fluorescence reading of each well with the fluorescence of the negative control wells (no fucoidan or virus).

The expression of the reporter gene, used as a proxy for successful transduction within each well, was then quantified using the luciferase assay. The media in the plate wells was replaced with 25 µL of DMEM and 25 µL of Bright-Glo^TM^ reagent (Promega, Madison, WI, USA). After 15 min, the luminescence signal was measured on the CLARIOStar plate reader. Luciferase readings were first normalized to resazurin fluorescence (cell viability), then expressed as a percentage of the virus-only control after subtraction of the no-virus background.

#### 4.3.3. Antifungal Activity

Cultures of *Candida auris* (NCPF 8978), *Candida dubliniensis* (NCPF 3949) and *Cryptococcus neoformans* (ATCC H99) were prepared by the addition of a single colony from a stock plate to 5 mL of YPD 1% yeast extract (Difco, BD Biosciences, San Jose, CA, USA), 2% peptone (Gibco, Thermo Fisher Scientific, Waltham, MA, USA), 2% dextrose (Formedium, Swaffham, UK) and 1% Uridine and 1% Adenine (Sigma-Aldrich, St. Louis, MO, USA) and incubated at 30 °C with shaking at 200 rpm for 24 h.

The antifungal bioactivity of the commercial extracts was assessed through a growth curve assay. Briefly, to a 48-well plate, 500 µL of supplemented YPD containing 5 mg/mL of each commercial fucoidan was inoculated with one of the three fungal strains at a cell density of 6 × 10^4^ cells per mL. Also included in the plate were a negative control (YPD inoculated with pathogens only), a blank (YPD only) and a control (YPD with fucoidan only) to ensure that increases in OD_600_ were a result of fungal growth and not aggregation effects or changes in turbidity.

The plate was incubated in a CLARIOStar plate reader (BMG Labtech, Ortenberg, Germany) at 30 °C for 24 h using a double orbital shake of 400 rpm. The absorbance of each well at 600 nm (OD_600_) was determined every hour. The blank data served to ensure no contamination had occurred, and a growth curve was prepared by subtracting the mean absorbances of the blank at each time point from the other wells before the mean values of each condition were determined. An antifungal inhibition was calculated by dividing the optical density measurement of the sample by that of the no fucoidan control at the end of the experimental run (24 h).

#### 4.3.4. Antibacterial Activity

Cultures of *Klebsiella pneumoniae* (Ecl8 derived from NCIB 418) [129] were prepared by adding a single colony selected from a stock plate to 5 mL of Luria–Bertani (LB) broth (Sigma-Aldrich, St. Louis, MO, USA) before being incubated at 37 °C under shaking for 24 h.

The antibacterial properties of each extract were assessed by an antimicrobial susceptibility assay based on the protocol set out by [130]. Briefly, overnight bacterial cultures were diluted to prepare a suspension containing approximately 10^3^ CFU/mL in PBS. 5 µL of the bacterial suspension was added to 1 mL of tryptic soy broth (TSB) (Sigma-Aldrich, St. Louis, MO, USA) containing 2 mg/mL and 5 mg/mL of commercial fucoidan. The tubes were then incubated at 37 °C for 1 h whilst shaking at 200 rpm using a ThermoMixer C (Eppendorf, Hamburg, Germany). After incubation, 100 µL of culture was spread on LB agar plates (Sigma-Aldrich, St. Louis, MO, USA) and incubated at 37 °C for 24 h. The number of colonies on each plate was counted, and the CFU/mL was calculated. Finally, the relative percentage of *K. pneumoniae* after exposure to fucoidan was determined and expressed as a percentage of the colony count of bacteria not exposed to any fucoidan sample.

#### 4.3.5. Prebiotic Activity

Cultures of *Lactobacillus casei* (ATCC 393) were prepared by inoculating 5 mL of Brain Heart Infusion (BHI) broth (Sigma-Aldrich, St. Louis, MO, USA) with a single colony selected from a stock plate. The culture was then incubated at 37 °C under shaking for 24 h.

The prebiotic activity was assessed using a similar assay to that used in the assessment of antifungal activity. A 96-well plate containing BHI broth was spiked with fucoidan at varying concentrations (5 mg/mL–1 µg/mL) and inoculated with *Lactobacillus casei* so that the OD_600_ value of the mixture was 0.05. Also included in the plate were a negative control (BHI inoculated with *L. casei* only), a blank (BHI only) and a control (BHI with fucoidan only) to ensure that increases in OD_600_ were a result of bacterial growth and not aggregation effects or changes in turbidity.

The plate was incubated at 37 °C for 48 h in a CLARIOStar plate reader (BMG Labtech, Ortenberg, Germany) with 400 rpm double orbital shaking. The absorbance of each well at 600 nm (OD_600_) was determined every hour, allowing a growth curve to be plotted. A prebiotic effect score was calculated by dividing the optical density measurement of the sample by that of the no fucoidan control at the end of the experimental run (48 h).

### 4.4. Statistical Analysis and Pearson’s Correlation Matrix

Data on the chemical, structural and biological activity of commercial extracts were statistically analyzed in Prism 10 (GraphPad) using one-way ANOVA with post hoc Tukey HSD test. Correlations between each biological activity (antioxidant, antiviral, antifungal, and prebiotic) were analyzed in RStudio version 4.1.1. and a Pearson correlation matrix generated using “corrplot” [23].

## 5. Conclusions

Fucoidan chemical structure and composition are key determinants in the biological activity an extract may possess. However, the relationship between these areas is poorly established, limiting the application of the extracts as a biomedical treatment. So far, attempts to understand these relationships have been limited to qualitative comparisons of chemical and biological datasets or testing of chemically modified fucoidans.

This study employed a correlation analysis to identify fucoidan structural and chemical characteristics potentially important for fucoidans to possess specific biological activities, worthy of further exploration. Four commercial extracts were chemically characterized before undergoing bioactivity testing (antioxidant, antiviral, antifungal and prebiotic activities). With antioxidant activity properties, antiviral inhibition of Nipah virus, antifungal activity against *C. dubliensis*, and a prebiotic effect on *L. casei* were observed. Contrastingly, despite reports within the literature, no antifungal effect on *Candida auris, Cryptococcus neoformans* or antibacterial activity against *Klebsiella pneumoniae* at our tested doses was observed in this study. These findings demonstrate the breadth of fucoidans’ bioactivity. 

The correlation analysis identified several potentially interesting chemical characteristics for bioactivity for further exploration. With a high fucose content, the primary monosaccharide, and a high sulfate content are suggested to enhance all bioactivities tested, supporting the view of existing literature. Furthermore, the correlation analysis appeared to address a conflict in the literature on the effect of molecular weight, suggesting that the optimal molecular weight varies depending on the specific bioactive application. The results suggest that large molecular weights are necessary for antifungal and prebiotic activities, while smaller ones are optimal for antiviral and antioxidant applications. In addition, high fucose content was identified as being beneficial for all activities, suggesting fucoidan has potential as a bioactive substance.

To strengthen these findings, this approach should be applied more widely to study the effects of chemically characterized fucoidans on a wider variety of microbes (viruses, bacteria, fungi), to give greater confidence in the correlations drawn in this study.

To further explore and verify the sulfate–function relationship identified, highly sulfated fucoidans should be produced using anion-exchange chromatography, and fucoidans of varying molecular weight should be created using membranes or size-exclusion chromatography. These fucoidans should then be tested in high-throughput assays to determine the effects of these extract characteristics. Mechanistic studies involving microbial knockouts and antibody-based antiviral assays should also be employed to examine how sulfate content and molecular weight affect fucoidans’ bioactivity.

## Data Availability

The original contributions presented in this study are included in the article/Supplementary Material. Further inquiries can be directed to the corresponding author.

## Supplementary Materials

The following supporting information can be downloaded at: https://www.mdpi.com/article/10.3390/molecules31101618/s1, including Antifungal Activity Spotting Assay; Antifungal Activity Spotting Assay Results; Elemental Analysis Raw Data (CHNS and ICP-OES); Monosaccharide Profile of Extracts (Absolute Terms); Molecular Weight Analysis (SEC-MALS); Prebiotic Growth Curves [34,131,132,133].
