# Supplementary material for: Structure-Function Correlations of Commercial Fucoidan Extracts: Antioxidant, Antiviral, Antifungal, Antibacterial and Prebiotic Activities
Source: Molecules. 2026 May 11;31(10):1618. doi: 10.3390/molecules31101618 (PMC13209295; doi:10.3390/molecules31101618)
Supplement: Supplementary file 1 [file molecules-31-01618-s001.zip › molecules-4259847-supplementary.pdf]

Supporting Information

Structure-Function Correlations of Commercial Fucoidan Extracts: Antioxidant, Antiviral, Antifungal, Antibacterial and Prebiotic Activities

Matthew Chadwick [1], Maria Sole Regina Lancerin [2], Patricia Hazelton [1], Kyriakos Vidalis [2], Emmanuel Petit [3], Paolina Lukova [4], Cédric Delattre [5,6], Xianfeng Chen [1], Thamarai Schneiders [2], Vasso Makrantoni [2,7], Richard Sloan [2,7] and Simone Dimartino [1,8,*]

[1] Institute for Bioengineering, The School of Engineering, The University of Edinburgh, Edinburgh, EH9 3JL, UK
[2] Centre for Inflammation Research, Institute of Regeneration and Repair, The University of Edinburgh, Edinburgh, EH16 4UU, UK
[3] UMRT INRAe 1158 BioEcoAgro-BIOPI, IUT-GB, Université de Picardie Jules Verne, 80025 Amiens, France
[4] Department of Pharmacognosy and Pharmaceutical Chemistry, Faculty of Pharmacy, Medical University of Plovdiv, Vasil Aprilov Str. 15A, 4002 Plovdiv, Bulgaria
[5] Université Clermont Auvergne, Clermont Auvergne INP, CNRS, Institut Pascal, 63000 Clermont-Ferrand, France
[6] Institut Universitaire de France (IUF), 1 Rue Descartes, 75005 Paris, France
[7] Zhejiang University-University of Edinburgh Institute, Zhejiang University School of Medicine, Zhejiang University, Haining, Zhejiang 314400, China
[8] Planet Crafting Labs, Edinburgh, EH10 4AX
* Correspondence: simone.dimartino@ed.ac.uk

S1. Experimental Section **1**
S1.1. Antifungal Activity Spotting Assay 1
S1.2. Antifungal Activity Spotting Assay Results 1
S2. Supporting Tables **2**
S2.1. Elemental Analysis Raw Data (CHNS & ICP-OES) 2
S2.2. Monosaccharide Profile of Extracts (Absolute Terms) 3
S3. Supporting Figures **4**
S3.1. Molecular Weight Analysis (SEC-MALS) 4
S3.1.1. Shandong 4
S3.1.2. Mark Nature 5
S3.1.3. Marinova 6
S3.1.4. ApexBio 7
S3.2. Prebiotic Growth Curves 8

S1. Experimental Section

S1.1. Antifungal Activity Spotting Assay

Preliminary tests of antifungal activity of fucoidan were performed using a serial dilution-based assay. Several fungal strains were used in this assay including *Candida albicans* (SC5314) [131], *Candida albicans* (BWP17) [132], *Candida glabrata* (NCPF 3831), *Candida parapsilosis* (NCPF 8384), *Candida dubliniensis* (NCPF 3949), *Candida auris (*NCPF 8978*)* and *Cryptococcus neoformans* (H99) [133]. Cultures were prepared by adding a single colony selected from a stock plate to 5mL of YPD media (1% yeast extract, 2% peptone, and 2% dextrose, 1% uridine, 1% adenine) before incubation at 30°C overnight.

The optical density at 600 nm of the cultures was determined using a SmartSpec Plus spectrophotometer (Bio-Rad Laboratories) and diluted in YPD broth (Sigma-Aldrich, St. Louis, MO, USA) supplemented with 1% yeast extract (Difco, BD Biosciences, San Jose, CA, USA), 2% peptone (Gibco, Thermo Fisher Scientific, Waltham, MA, USA), 2% dextrose (Formedium) and 1% Uridine and 1% Adenine (Sigma-Aldrich, St. Louis, MO, USA) to a colony count of 3 × 10^7 CFU/mL (OD_{600} of 1). A six-point dilution series of these cultures was prepared using a dilution factor of 5. 3 µL of each dilution was spotted onto YPD agar plates (Difco, BD Biosciences, San Jose, CA, USA) spiked with 2 mg/mL or 5 mg/mL of Shandong fucoidan, or no fucoidan. The plates were then incubated at 30°C for 24 and 48 hours and examined for evidence of inhibition of bacterial growth.

S1.2. Antifungal Activity Spotting Assay Results

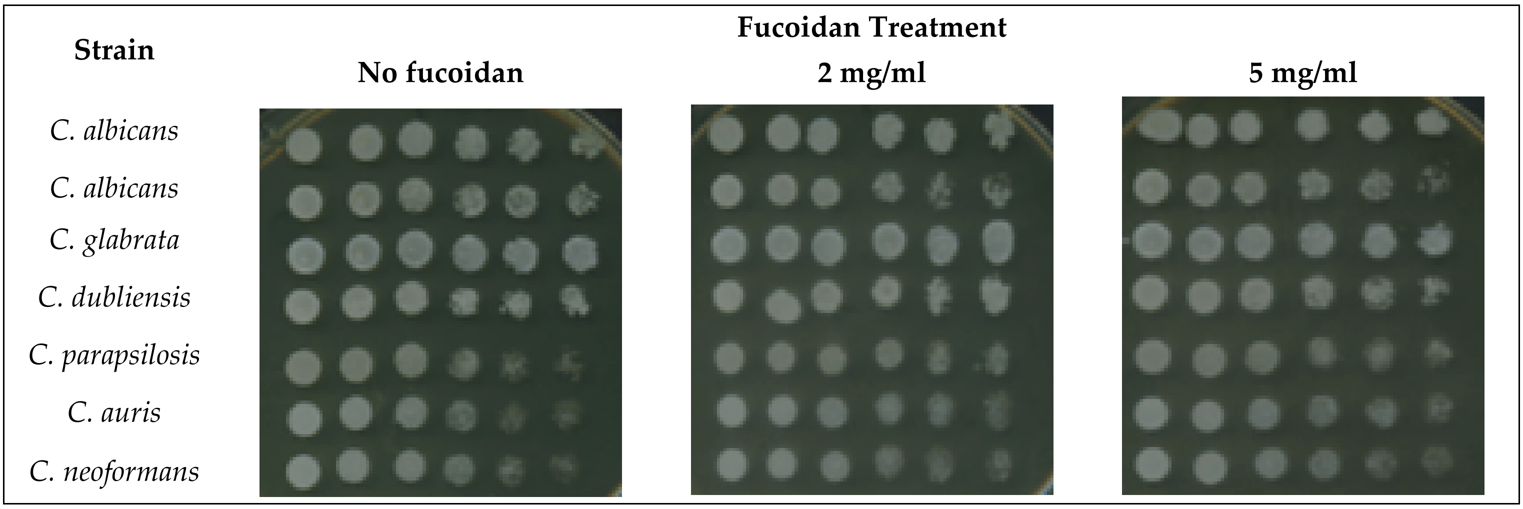

Figure S1. *Preliminary antifungal tests carried out by spotting assay using Shandong fucoidan using Candida albicans (SC5314) [131], Candida albicans (BWP17) [132], Candida glabrata (NCPF 3831), Candida dubliniensis (NCPF 3949), Candida auris (NCPF 8978)* and *Cryptococcus neoformans (H99) [133].*

The antifungal spotting assay showed very little inhibition of the majority of fungal strains in the presence of Shandong fucoidan at any concentration. However, at 5 mg/mL, a slight inhibition of *Candida dubliensis* was observed at the lowest dilution (last column) as shown by a reduction in colonies present compared to the no fucoidan control. This warranted further study through growth curves (section 2.4.3) in the main manuscript.

S2. Supporting Tables

S2.1. Elemental Analysis (CHNS and ICP-OES)

Table S1. *Elemental analysis of four commercial fucoidans (Shandong, Mark Nature, Marinova, ApexBio), obtained through CHNS and ICP-OES analysis. Both analyses were carried out in triplicate (n=3), negative values indicate signal was below detection limits. Data reported as mean ± SD. Different letters in the same row indicate a statistical difference between samples using a 95% confidence interval.*

Sample	CHNS Elemental Analysis				ICP-OES
	Carbon (%)	Hydrogen (%)	Nitrogen (%)	Sulphur (%)	Sulphur (%)
Shandong	29.747±2.527[b]	5.050±0.413[b]	0.817±0.068[a]	11.434±1.171[a]	6.678±0.111[b]
Mark Nature	36.820±0.493[a]	5.731±0.107[b]	0.241±0.009[c]	2.060±0.071[b]	2.115±0.059[c]
Marinova	28.564±0.223[a]	4.534±0.027[c]	0.532±0.005[b]	11.156±0.062[a]	7.219±0.026[a]
ApexBio	34.631±0.605[b]	6.658±0.135[a]	0.176±0.004[c]	-0.300±0.025[c]	0.057±0.004[d]

Table S2 presents raw elemental CHNS and ICP-OES data for each of the four commercial fucoidans. As can be seen the ApexBio sample's sulphur content was below the detection limit of the CHNS analysis therefore to obtain an accurate measurement of sulphur content for this extract a follow up ICP-OES analysis was carried out. This data was then used to calculate the sulphation degree and protein content (Table 1) according to Zayed *et al.* [34] using Equations 1 and 2:

$$Sulphation\ Degree = \frac{\left(\frac{S(\%)}{32}\right)\cdot 6}{\left(\frac{C(\%)}{12}\right)} \qquad (1)$$

Where C(%) and S(%) and the samples relative carbon and sulphur contents, 12 and 32 are the molecular weights of carbon and sulphur elements. With 6 being the number of carbon atoms per sugar monomer (assuming all monomers are hexoses). Note for ApexBio the S(%) used was from ICP-OES to estimate sulphation degree.

$$Protein\ Content = N(\%) \cdot 6.25 \qquad (2)$$

Where N(%) is the relative nitrogen content of a sample, and 6.25 is a constant (assuming that 16% if each protein is nitrogen).

S2.2. Monosaccharide Profile of Extracts

Table S2. *Monosaccharide profiles of commercial fucoidan samples obtained through HPAEC-PAD expressed in absolute values (μg/10 mg). Analysis carried out in triplicate (n=3). Data reported as mean ± SD. Different letters in the same row indicate a statistical difference between samples using a 95% confidence interval.*

	**Sample**	**Shandong**	**Mark Nature**	**Marinova**	**ApexBio**
Monosaccharides	Fucose (μg/10 mg)	296.682±18.697	176.385±46.802	317.557±47.311	0.000±0.000
	Glucose (μg/10 mg)	45.251±3.172	1412.268±80.135	17.66±4.551	0.000±0.000
	Mannitol (μg/10 mg)	3.262±0.000	0.000±0.000	0.000±0.175	465.702±12.108
	Arabinose/Rhamnose (μg/10 mg)	21.837±0.000	9.465±2.075	0.000±3.986	0.000±0.000
Uronic Acids	Glucuronic Acid (μg/10 mg)	187.872±4.479	45.173±15.496	29.156±22.066	0.000±0.000
	Guluronic Acid (μg/10 mg)	0.000±1.152	24.354±6.362	1.152±0.000	0.000±0.000
	Mannuronic Acid (μg/10 mg)	8.545±3.547	264.338±43.691	59.754±4.575	0.000±0.000
Unknowns	Unknown 1 (μg/10 mg)	25.691±1.53	46.12±2.781	12.819±2.059	0.000±0.000
	Unknown 2 (μg/10 mg)	20.083±0.495	27.706±10.044	0.000±0.000	0.000±0.000
	Unknown 3 (μg/10 mg)	112.099±6.091	292.925±14.701	64.085±14.818	0.000±0.000
	Unknown 4 (μg/10 mg)	58.878±1.374	12.023±8.805	37.835±10.548	0.000±0.000
	Total (μg/10 mg)	780.200	2046.724	804.052	465.702

Four unknowns were detected in HPAEC-PAD analysis; these are believed to be impurities or partially polysaccharides. The Monosaccharide and Uronic acid contents were converted to relative percentages by dividing the absolute concentration of each monosaccharide/uronic acid by the total mass detected in the sample, including unknowns.

S3. Supporting Figures

S3.1. Molecular Weight Analysis (SEC-MALS)

S3.1.1. Shangdon

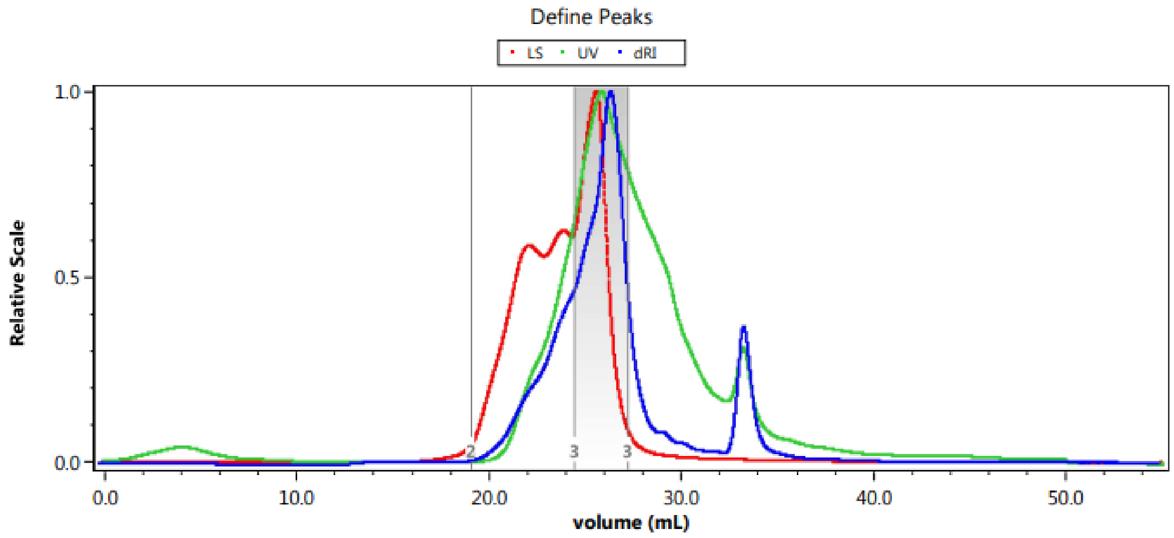

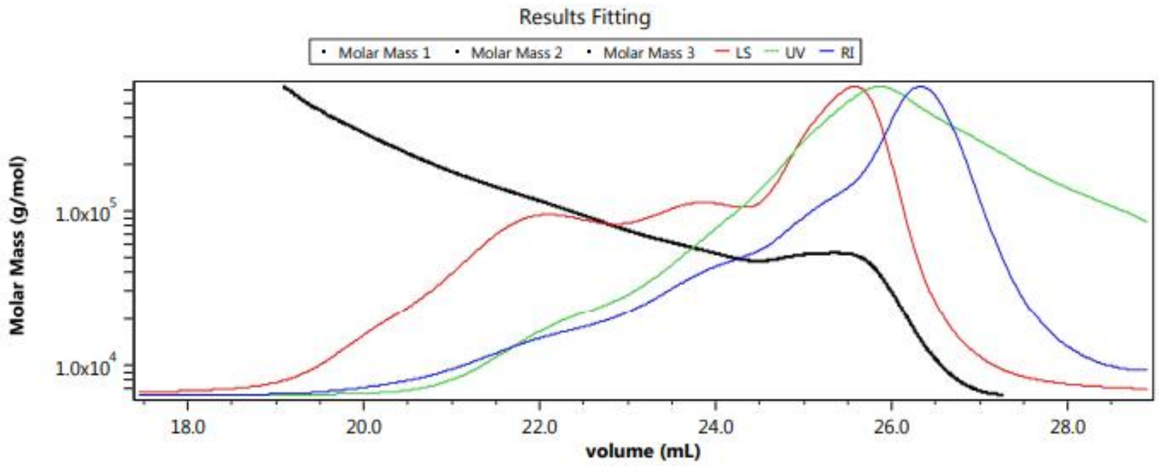

	Peak 1	Peak 2	Peak 3
**Peak Settings**			
Peak Limits (mL)	19.100-27.260	19.100-24.500	24.500-27.260
Injected Mass (μg)	2000.00	2000.00	2000.00
Calculated Mass (μg)	898.18	297.47	600.71
Mass Recovery (%)	44.90	14.90	30.00
Mass Fraction (%)	100.00	33.10	66.90
**Calculated Molar Masses**			
Mn (kDa)	22.31±0.08	71.81±0.50	16.65±0.06
Mw (kDa)	49.46±0.33	88.88±0.61	30.01±0.19
Polydispersity (Mw/Mn)	2.22±0.02	1.24±0.01	1.80±0.01

S3.1.2. Mark Nature

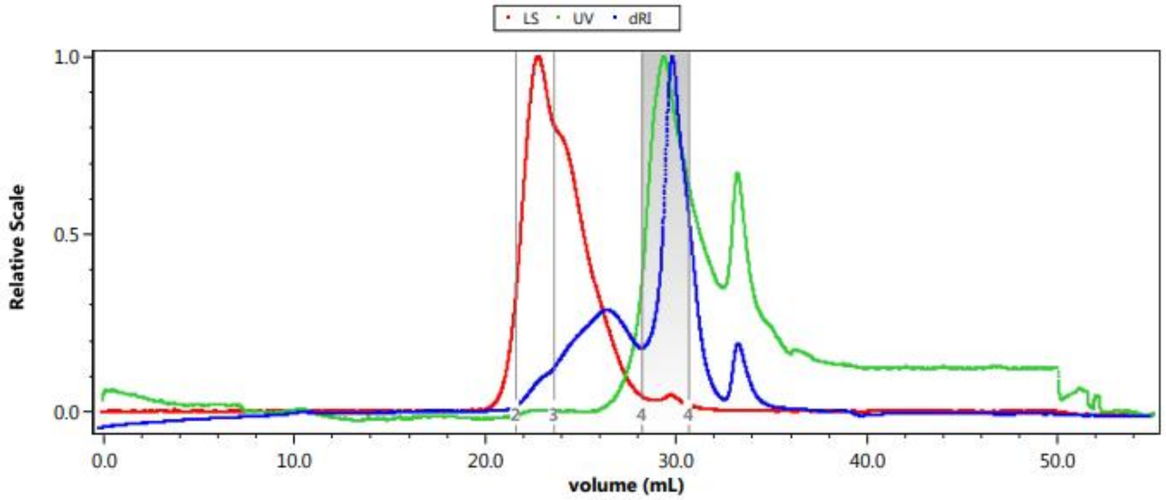

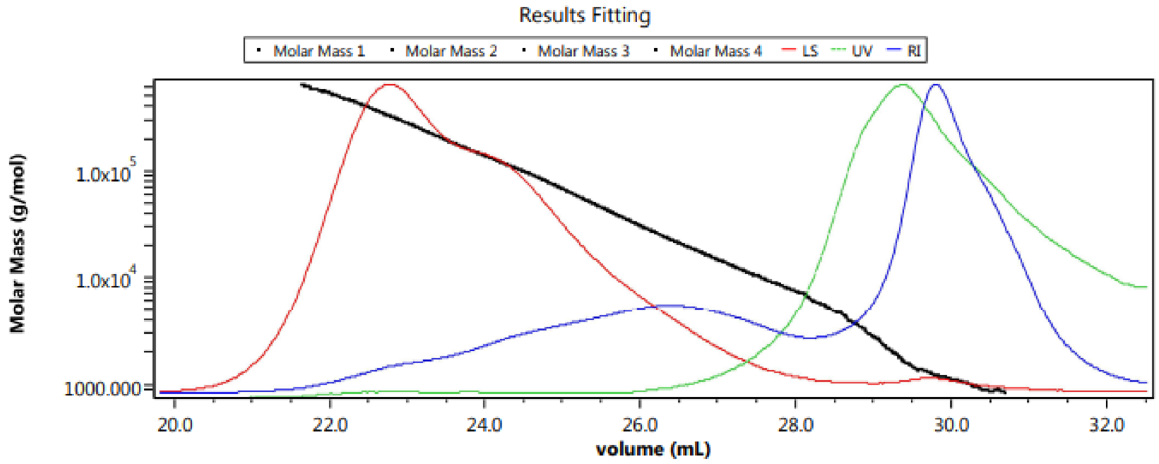

Peak Settings				
	Peak 1	Peak 2	Peak 3	
Peak Limits (mL)	21.640-30.700	21.640-23.600	23.600-28.200	28.200-30.700
Injected Mass (μg)	250.00	250.00	250.00	250.00
Calculated Mass (μg)	112.65	6.07	45.43	61.14
Mass Recovery (%)	45.1	2.4	18.2	24.5
Mass Fraction (%)	100.0	5.4	40.3	54.3
**Calculated Molar Masses**				
Mn (kDa)	2.30±0.08	284.00±1.73	21.78±0.27	1.31±0.05
Mw (kDa)	36.54±0.25	315.90±1.78	46.23±0.32	1.66±0.05
Polydispersity (Mw/Mn)	15.87±0.55	1.11±0.01	2.12±0.03	1.27±0.06

S3.1.3. Marinova

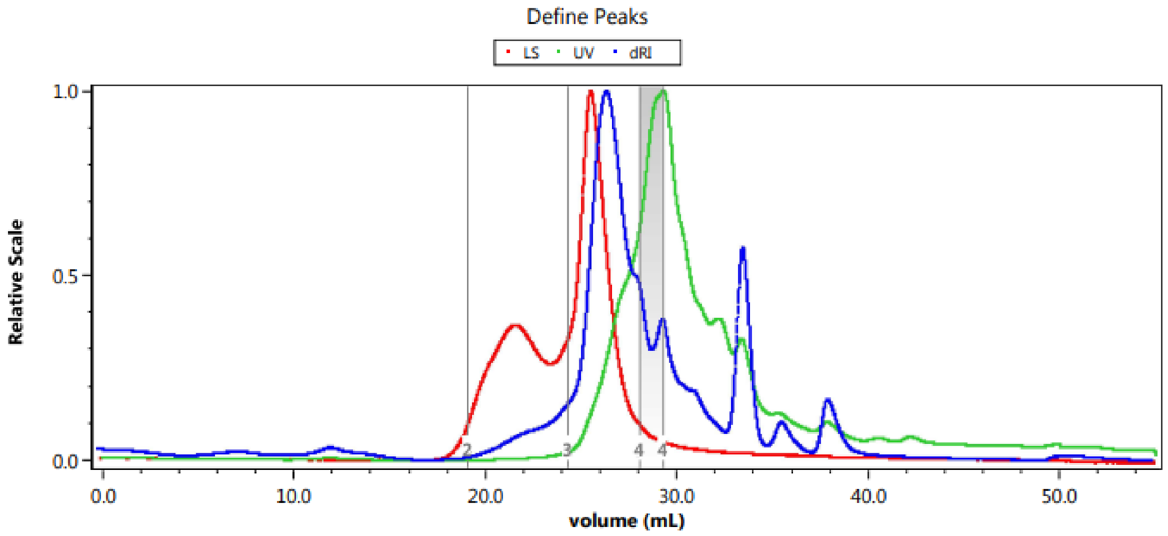

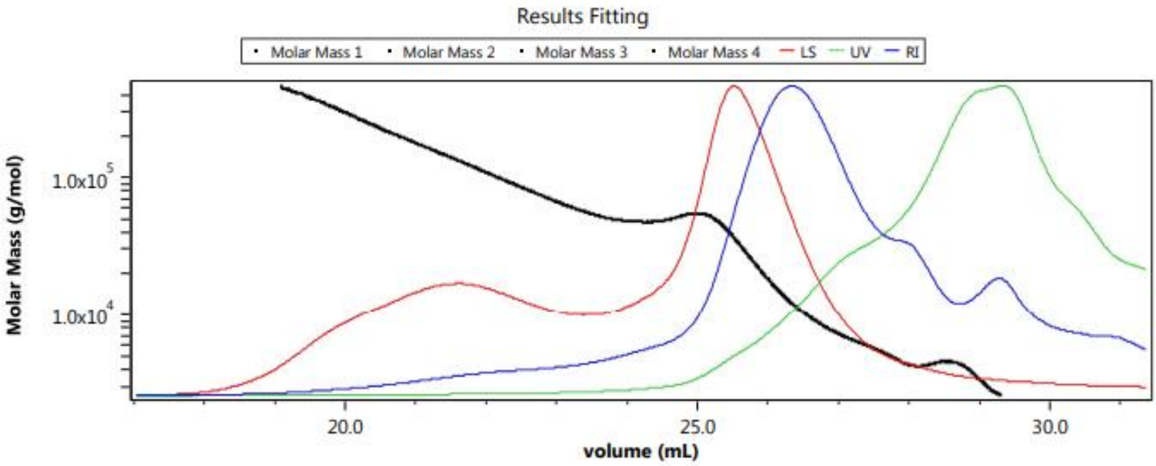

Peak Settings				
	**Peak 1**	**Peak 2**	**Peak 3**	**Peak 4**
Peak Limits (mL)	19.100-29.300	19.100-24.300	24.30-28.100	28.100-29.300
Injected Mass (μg)	2000.00	2000.00	2000.00	2000.00
Calculated Mass (μg)	372.16	40.22	280.24	51.71
Mass Recovery (%)	18.60	2.00	14.00	2.60
Mass Fraction (%)	100.0	10.80	75.30	13.90
**Calculated Molar Masses**				
Mn (kDa)	8.88±0.16	74.92±0.58	10.14±0.08	3.79±0.14
Mw (kDa)	24.91±0.23	102.5±0.94	17.65±0.15	3.91±0.13
Polydispersity (Mw/Mn)	2.81±0.06	1.37±0.02	1.74±0.02	1.03±0.05

S3.1.4. ApexBio

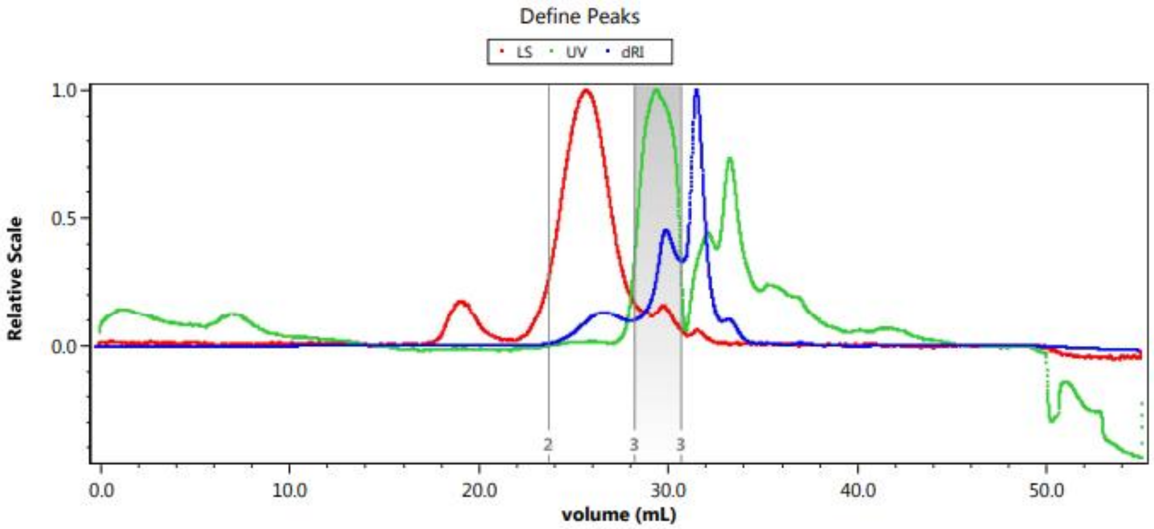

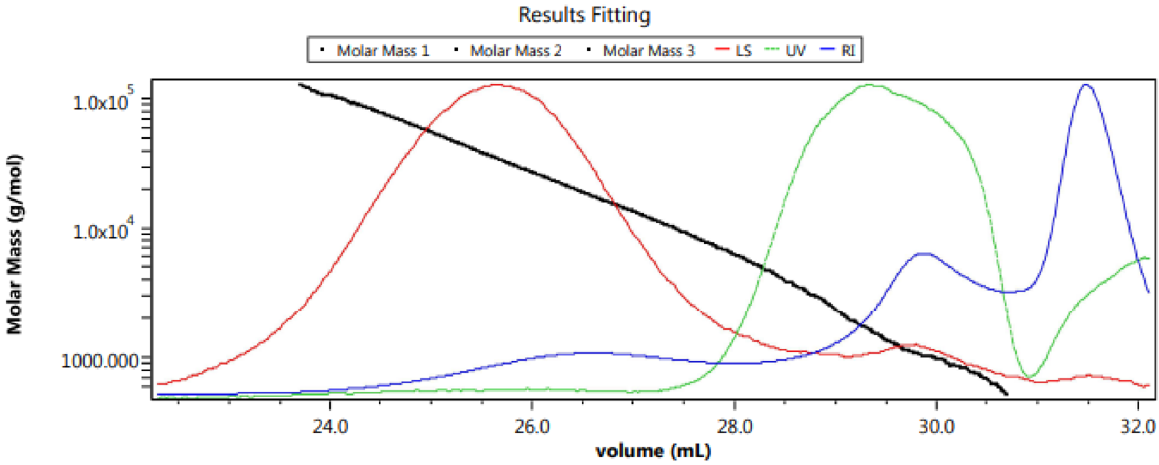

Peak Settings			
	**Peak 1**	**Peak 2**	**Peak 3**
Peak Limits (mL)	23.700-30.700	23.700-28.200	28.200-30.700
Injected Mass (µg)	250.00	250.00	250.00
Calculated Mass (µg)	120.77	42.35	78.42
Mass Recovery (%)	48.3-	16.90	31.40
Mass Fraction (%)	100.00	35.10	64.90
**Molar Masses**			
Mn (kDa)	1.61±0.05	15.03±0.18	1.09±0.03
Mw (kDa)	9.65±0.09	24.83±0.18	1.45±0.04
Polydispersity (Mw/Mn)	6.00±0.18	1.65±0.02	1.34±0.05

S3.2. Prebiotic Growth Curves

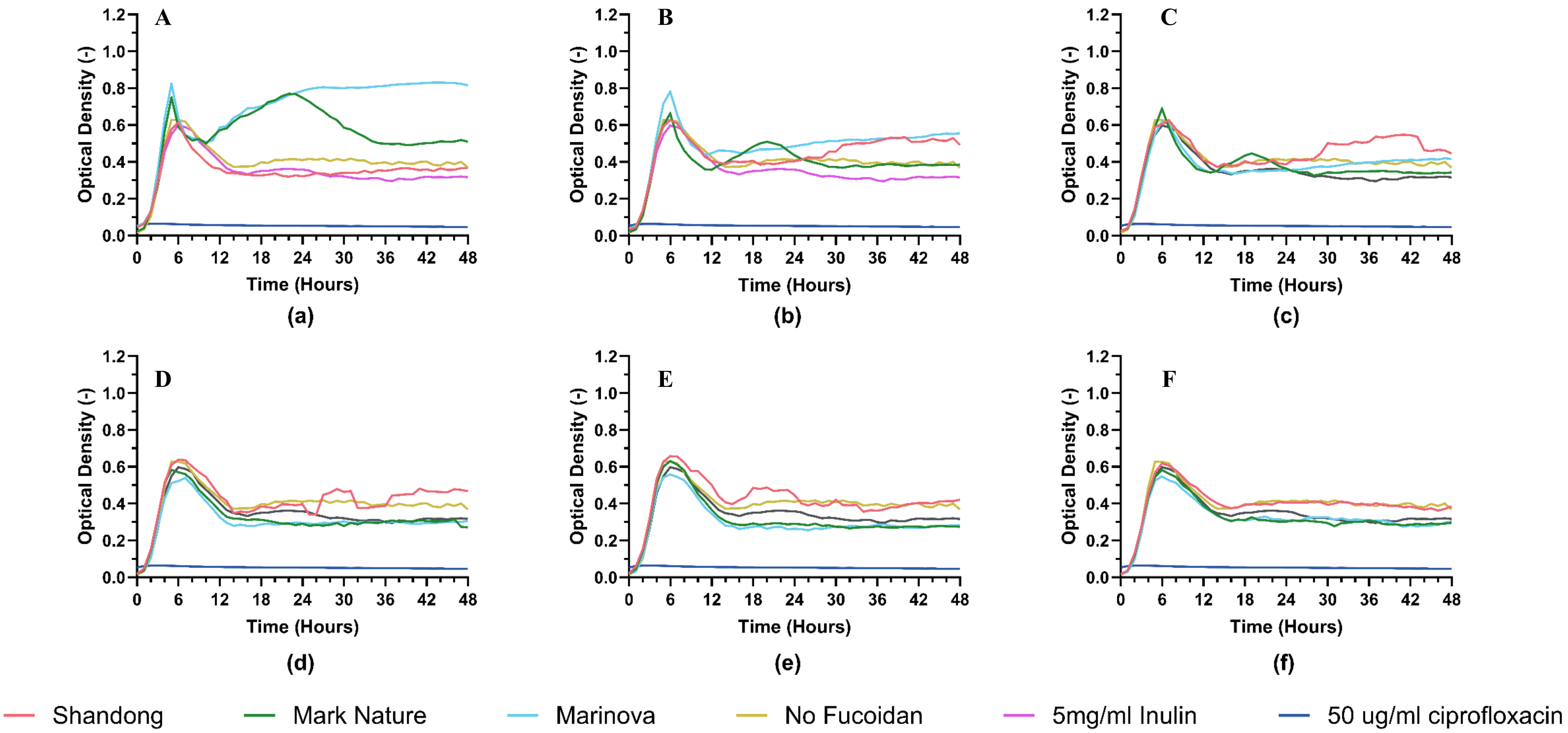

Figure S2. *Lactobacillus casei growth curves in the presence of commercial fucoidans at a concentration of* ***A.*** *5 mg/mL,* ***B.*** *2 mg/mL,* ***C.*** *1 mg/mL,* ***D.*** *100 µg/mL,* ***E.*** *10 µg/mL,* ***F.*** *1 µg/mL. n=3 for all test conditions.*

As can be clearly seen in Figure S2, the growth curves of *Lactobacillus casei* in the presence of the Mark Nature and Marinova extracts reach a greater OD_{600} compared to the no fucoidan control at 5 mg/mL, indicating a prebiotic effect in which bacterial growth is enhanced by these extracts. This effect reduces at 2 mg/mL, with the growth curves eventually overlapping at lower concentrations, suggesting a dose-dependence.
